# Sequential Pressure-Induced *B*1–*B*2 Transitions in the Anion-Ordered Oxyhydride Ba_2_YHO_3_

**DOI:** 10.1021/acs.inorgchem.2c00465

**Published:** 2022-04-22

**Authors:** Harry W. T. Morgan, Takafumi Yamamoto, Takumi Nishikubo, Takuya Ohmi, Takehiro Koike, Yuki Sakai, Masaki Azuma, Hirofumi Ishii, Genki Kobayashi, John E. McGrady

**Affiliations:** † Department of Chemistry and Biochemistry, University of California, Los Angeles, Los Angeles, California 90095-1569, United States; ‡ Department of Chemistry, University of Oxford, South Parks Road, Oxford OX1 3QR, United Kingdom; § Laboratory for Materials and Structures, Tokyo Institute of Technology, Yokohama, Kanagawa 226-8503, Japan; ∥ Kanagawa Institute of Industrial Science and Technology, Ebina 243-0435, Japan; ⊥ National Synchrotron Radiation Research Center, Hsinchu 30076, Taiwan; # Department of Materials Molecular Science, Institute for Molecular Science, 38 Nishigonaka, Myodaiji, Okazaki, Aichi 444-8585, Japan; ¶ SOKENDAI (The Graduate University for Advanced Studies), 38 Nishigonaka, Myodaiji, Okazaki, Aichi 444-8585, Japan

## Abstract

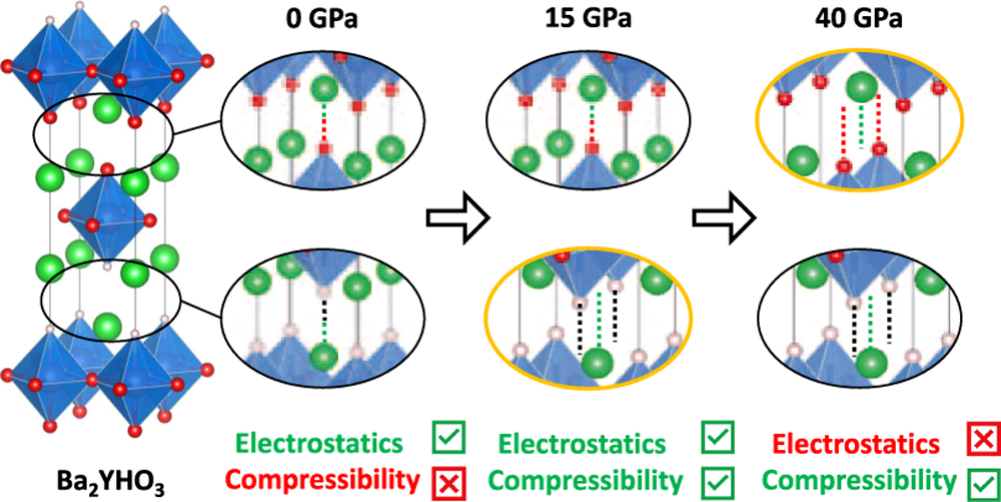

We present a detailed experimental and computational investigation of the influence of pressure on the mixed-anion oxyhydride phase Ba_2_YHO_3_, which has recently been shown to support hydride conductivity. The unique feature of this layered perovskite is that the oxide and hydride anions are segregated into distinct regions of the unit cell, in contrast to the disordered arrangement in closely related Ba_2_ScHO_3_. Density functional theory (DFT) calculations reveal that the application of pressure drives two sequential *B*1–*B*2 transitions in the interlayer regions from rock salt to CsCl-type ordering, one in the hydride-rich layer at approximately 10 GPa and another in the oxide-rich layer at 35–40 GPa. To verify the theoretical predictions, we experimentally observe the structural transition at 10 GPa using high-pressure X-ray diffraction (XRD), but the details of the structure cannot be solved due to peak broadening of the XRD patterns. We use DFT to explore the structural impact of pressure on the atomic scale and show how the pressure-dependent properties can be understood in terms of simple electrostatic engineering.

## Abstract 2

We investigate a sequence of pressure-induced phase transitions in Ba_2_YHO_3_, a perovskite oxyhydride with a unique layered anion ordering. Density functional theory and X-ray diffraction together provide a detailed and informative picture of the changes to the crystal structure across the pressure range. This work provides new insights into nonuniform structural flexibility in 2D materials, which can aid targeted materials design in other chemical systems.

## Introduction

**Figure 1:**
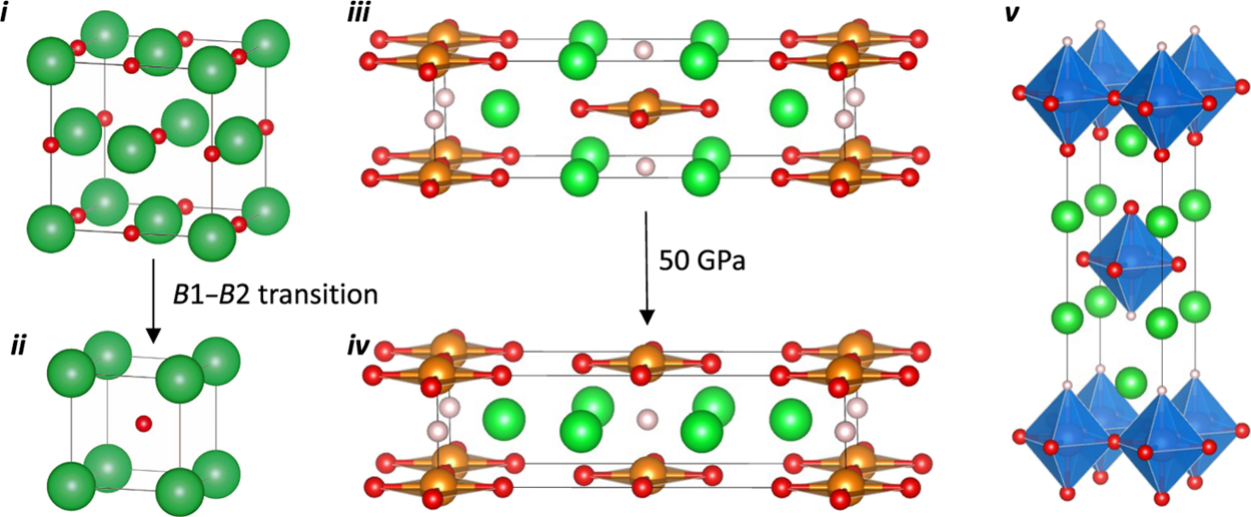
Ionic materials relevant to the *B*1–*B*2 transition in RP phases. (i) SrO in the ambient-pressure rock salt (*B*1) structure. (ii) SrO in the high-pressure CsCl (*B*2) structure. (iii) Ambient-pressure structure of Sr_2_VO_3_H. (iv) High-pressure structure of Sr_2_VO_3_H, having undergone the *B*1–*B*2 transition. (v) Ambient-pressure unit cell of Ba_2_YHO_3_. Sr and Ba atoms are shown in green, V in orange, Y in blue, H in white, and O in red.

High-pressure studies on inorganic materials, both experimental and theoretical, continue to provide insights into structure and bonding unobtainable under standard laboratory conditions. The most obvious application of such techniques is in deep-earth chemistry, where pressures may reach 360 GPa, and great interest has arisen recently in the high-temperature superconducting properties of hydrides at high pressures.^1−3^ However, recent work on low-dimensional materials has shown that such extreme conditions are not always required to induce substantial structural changes. A well-studied example is the “*B*1–*B*2” transition in binary oxides and halides, where the ion ordering changes from six-coordinate rock salt to eight-coordinate CsCl (Figure 1i,ii).^4−7^ Closely related phenomena are seen in many of the Ruddlesden–Popper (“RP”) phases, a diverse family of solids with alternating perovskite and rock salt layers. These can undergo a pressure-induced structural transition akin to the *B*1–*B*2 transition observed in binary materials, in which the perovskite layers shift to change the interlayer spacing from rock salt ordering to CsCl ordering (Figure 1iii, iv). This transition is common to A_*n*+1_B_*n*_O_3*n*+1_ RP phases (Sr_3_Ir_2_O_7_),^8^ anion-deficient analogues (Sr_2_CuO_3_ and Sr_3_Fe_2_O_5_),^9,10^ and oxyhydrides (Sr_2_VO_3_H and Sr_3_V_2_O_5_H_2_),^11^ and the critical pressure has been shown to depend on the radius ratio of the A cation and the anion, *R*_A_/*R*_X_. Complex relationships between the *B*1–*B*2 transition and electronic properties have been studied in *d*^*n*^ metal systems, with Sr_3_Fe_2_O_5_ and Sr_3_Ir_2_O_7_ undergoing transport transitions near the *B*1–*B*2 critical pressure,^8,9,12,13^ while the structural transition inhibits an insulator–semimetal transition in the vanadium oxyhydride family.^11,14^

**Figure 2:**
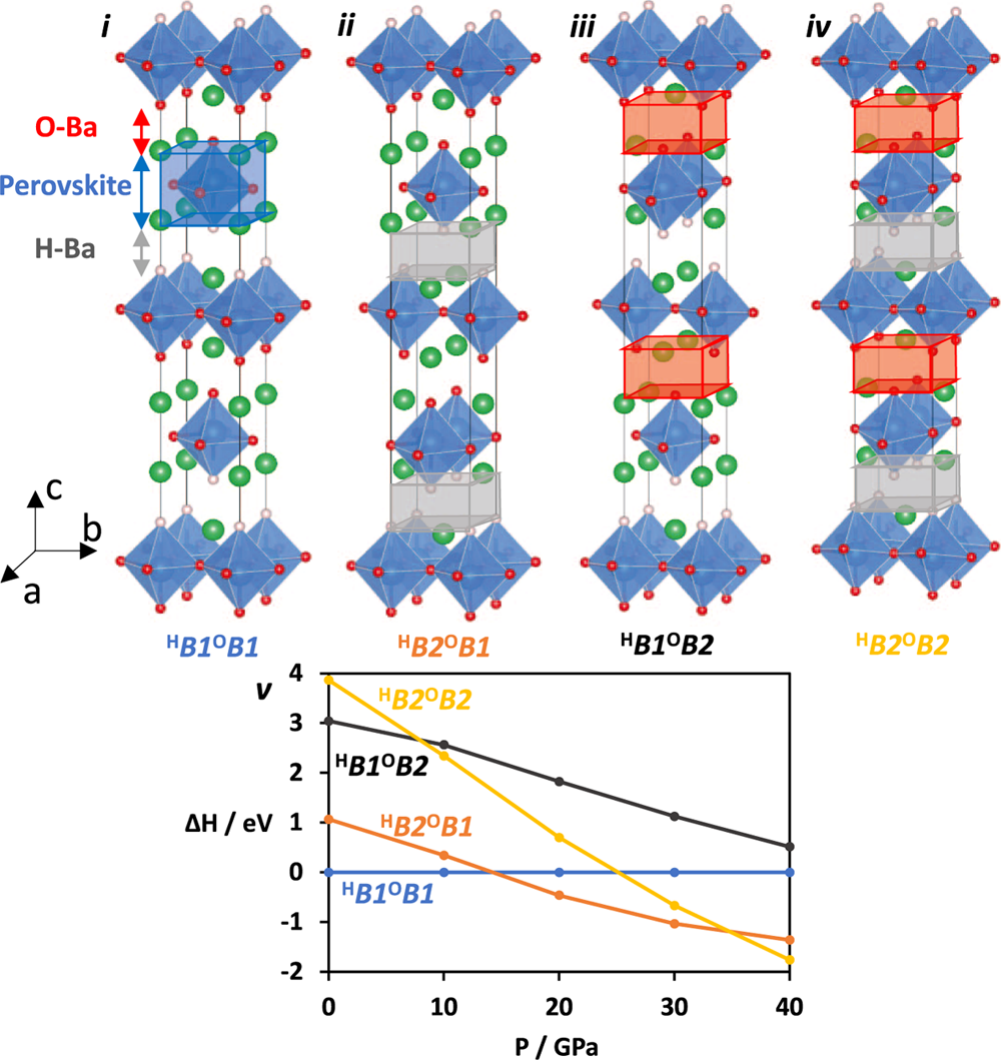
High-pressure geometries of Ba_2_YHO_3_ with Ba in green, Y in blue, H in white, and O in red. (i–iv) Unit cells generated by *B*1–*B*2 transitions in regions of the cell as indicated. Red and gray boxes mark regions in which the ordering has been changed by a local transition. (v) Enthalpies relative to the ^H^*B*1^O^*B*1 cell as a function of pressure.

Anion ordering in mixed-anion perovskites is finely balanced, with small changes to the cations able to dramatically alter the anion distribution.^15^ RP oxyhydrides contain two distinct anion sites, one contained in the perovskite layer and one in the “rock salt” layer (Figure 2i); the distribution of anions over these sites is dictated by electrostatics and the electronic configuration of the B cation. Sr_2_VO_3_H has hydrides in the perovskite layer such that each V^3+^ center has two mutually trans H^–^ ligands, an unusual arrangement that appears to be unique to *d*^2^ metals.^15,16^ Our previous work showed that the high-pressure behavior of Sr_2_VO_3_H is governed by the incompressibility of the V–O bonds in the perovskite layer, forcing more dramatic structural changes to the V–H bonds and to the rock salt layers, where the *B*1–*B*2 transition is localized.^11^

Ba_2_ScHO_3_ and Ba_2_YHO_3_ (Figure 1v), both recently synthesized and studied by our groups,^17−20^ are chemically simpler in that they have *d*^0^ B cations, so their structural preferences are controlled by electrostatics. Oxide and hydride anions are distributed across the available sites in accordance with the principle that the most charged anion coordinates to the most charged cation, so O^2–^ coordinates to Sc/Y^3+^ in the perovskite layer and H^–^ coordinates to Ba^2+^ in the rock salt layer. Ba_2_ScHO_3_ and Ba_2_YHO_3_ differ in that the rock salt sites in Ba_2_ScHO_3_ are occupied in a disordered fashion by hydride and oxide anions in a 1:1 ratio, while those in Ba_2_YHO_3_ are segregated into alternating all-oxide and all-hydride layers. This ordering difference can be explained by the Goldschmidt tolerance factor, which shows that the ionic radii of Ba_2_YHO_3_ deviate from those required for a cubic perovskite. Such deviations can result in various types of structural distortion, from tilting of the BO_6_ octahedra to inclusion of face-sharing octahedral pairs alongside the usual corner-sharing arrangement. In Ba_2_YHO_3_, the size mismatch leads to a reduction of symmetry via anion ordering.^18^ The remarkable ordering pattern presents the possibility of two distinct “*B*1–*B*2” transitions at elevated pressures, one localized in the hydride-rich layer and the other in the oxide-rich layer. As a result, there may be an intermediate region in which half of the unit cell adopts CsCl ordering, while the other half retains rock salt ordering. The primary motivation for studying these materials is their ability to act as hydride anion conductors, and a previous computational study has shown that the principal anion conduction mechanism in Ba_2_ScHO_3_ changes under pressure, from an interstitial-mediated pathway to one involving vacancies^19^ via a “bottleneck” transition state that has been observed in other hydride conductors.^21,22^ The identification of materials with new hydride ordering patterns is, therefore, an important step in the study of novel hydride-ion conductors.

In this study, we present high-pressure diffraction studies that provide clear evidence for a phase transition at 10 GPa and rather less conclusive data that support the possibility of a further transition between 35 and 40 GPa. The diffraction patterns in the region between 10 and 35 GPa cannot be solved directly but are consistent with a theoretically predicted intermediate phase with CsCl ordering in the hydride-rich layer and rock salt ordering in the oxide layer. The broadening of the diffraction peaks means that atomic-level detail of the transitions is not accessible from the experiment, so we turn to density functional theory (DFT) to explore the pressure dependence of Ba_2_YHO_3_. The predicted diffraction patterns of the most stable phases are fully consistent with the experimental data, allowing us to probe the optimized structures for atomic-level details on the transitions that we observe in the experiment. A comparison with our previously reported results on Sr_2_VO_3_H offers some insights into the effects of dimensionality and electron configuration on these phase transitions.

## Methods

### Sample Preparation

Polycrystalline samples of Ba_2_YHO_3_ oxyhydride were synthesized using a solid-state reaction under high pressure and temperature using a cubic anvil-type cell. The starting materials of BaH_2_ (Mitsuwa Chemical, 99.5%), BaO (Aldrich, 99.99%), Y_2_O_3_ (Aldrich, 99.999%), and NaH (Aldrich, 95%) were weighed in an Ar-filled glovebox, thoroughly mixed in a planetary ball mill, and sealed in a NaCl capsule inside a pyrophyllite cell with a graphite heater. The cell was heated at 800 °C under 4 GPa for 1 h.

### High-Pressure X-ray Diffraction

The laboratory X-ray diffraction (XRD) profiles of Ba_2_YHO_3_ under high pressures were recorded by using Mo Kα radiation from a 5.4 kW Rigaku rotating anode generator equipped with a 100 μm collimator. Powder samples were loaded into a 140 μm hole of preindented rhenium gaskets of the diamond-anvil cells (DACs) with a 350 μm curet. Daphne oil 7373 was used as a pressure transmitting medium. The fluorescence shift of ruby was used to calibrate the pressure. To estimate the pressure distribution along the sample, several ruby chips were placed inside the hole at different distances from its center. It was found that the pressure gradient at the samples increases with pressure but would not exceed 5 GPa at maximum pressure. The diffracted X-rays were collected using an imaging plate. The synchrotron XRD profiles of Ba_2_YHO_3_ under high pressures were recorded on the BL12B2 beamline of SPring-8 with a wavelength of 0.61992 Å. Powder samples were loaded into a 140 μm hole of preindented stainless gaskets of DACs with a 400 μm curet. Daphne oil 7373 was used as a pressure transmitting medium.

### Quantum Chemical Calculations

DFT calculations were performed using VASP version 5.4.1.^23^ Projector-augmented wave pseudopotentials and the Perdew–Burke–Ernzerhof exchange–correlation functional were used,^24,25^ with a plane-wave energy cutoff of 600 eV. The Brillouin zone was sampled with a 7 × 7 × 1 *k*-point grid for all cells. A spin-restricted ansatz was used for all calculations. Enthalpies were calculated by adding the self-consistent field electronic energy to the product of the volume of the cell and the applied external pressure according to the standard equation
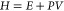


## Results and Discussion

### Prediction of Phase Transitions

We have identified four possible structures for Ba_2_YHO_3_ by imposing rock salt (*B*1) or CsCl (*B*2) ordering in the hydride-rich and oxide-rich layers, denoted H–Ba and O–Ba respectively, as indicated in Figure 2. To include all possibilities, we have doubled the *c* lattice parameter of the crystallographic unit cell shown in Figure 1v. The four cells are labeled according to the ion ordering (*B*1 or *B*2) in the H–Ba and O–Ba layers such that the observed structure at ambient pressure, where both H–Ba and O–Ba have *B*1 ordering, is denoted ^H^*B*1^O^*B*1. A *B*1–*B*2 transition localized in the H–Ba layer leads to ^H^*B*2^O^*B*1, while a transition in the O–Ba layer leads to ^H^*B*1^O^*B*2, and the cell with CsCl ordering in both layers is denoted ^H^*B*2^O^*B*2. These three structures, which are excited states under ambient conditions, can be generated from the ambient-pressure ^H^*B*1^O^*B*1 cell by shifting appropriate parts of the cell by *a*/2, as highlighted in Figure 2(i–iv). Rock salt ordering is favored by electrostatics as it brings oppositely charged ions into close contact, but these short distances render the structure incompressible. By contrast, CsCl ordering increases electrostatic repulsion but increases compressibility and will be enthalpically favored by *P*Δ*V* at high pressure. Phase transitions therefore occur when the reduction in volume outweighs the energetic cost of changing the anion ordering.

All four structures were fully optimized at pressures spanning 0–40 GPa; enthalpies relative to the ambient-pressure ^H^*B*1^O^*B*1 structure are shown in Figure 2v.

This phase diagram confirms that the ^H^*B*1^O^*B*1 structure is indeed the most stable at ambient pressure and that two independent phase transitions occur, one at approximately 15 GPa and a second at approximately 35 GPa. The first of these (to ^H^*B*2^O^*B*1) corresponds to a change to CsCl ordering in the H–Ba layer, while the second (to ^H^*B*2^O^*B*2) results in CsCl-file ordering throughout the structure. The fourth phase, ^H^*B*1^O^*B*2 (black line in Figure 2v, where only the oxide layer adopts CsCl ordering, is never the most stable and should, therefore, remain inaccessible to experiments across the entire pressure range. The calculations therefore identify a wide window spanning 20 GPa, where the intermediate phase with mixed rock salt/CsCl ordering should be accessible. The first transition pressure of 15 GPa is lower than the reported values for other RP phases^10,11^ and is accessible here only because the electrostatic term that favors rock salt ordering is much smaller in the hydride layer than in the oxide layer due to the smaller charge on H^–^. The same electrostatic trend is found in *B*1–*B*2 transitions in binary solids containing monovalent and divalent anions. The transition pressure is known to correlate with the ionic radius ratio, *R*_A_/*R*_X_, such that increasing the relative size of the cation causes the critical pressure to fall. However, BaO and KH have very similar radius ratios (0.96 and 0.99, respectively) but *B*1–*B*2 transition pressures of 15 GPa and 4 GPa, respectively, showing that the higher electrostatic energy of divalent BaO hinders the adoption of the CsCl structure^4,26^ just as it does in the O–Ba layer of Ba_2_YHO_3_. Additionally, H^–^ anions have been shown to be intrinsically more compressible than O^2–^ anions in studies on vanadium oxyhydrides.^11,14^

### High-Pressure Powder XRD

**Figure 3:**
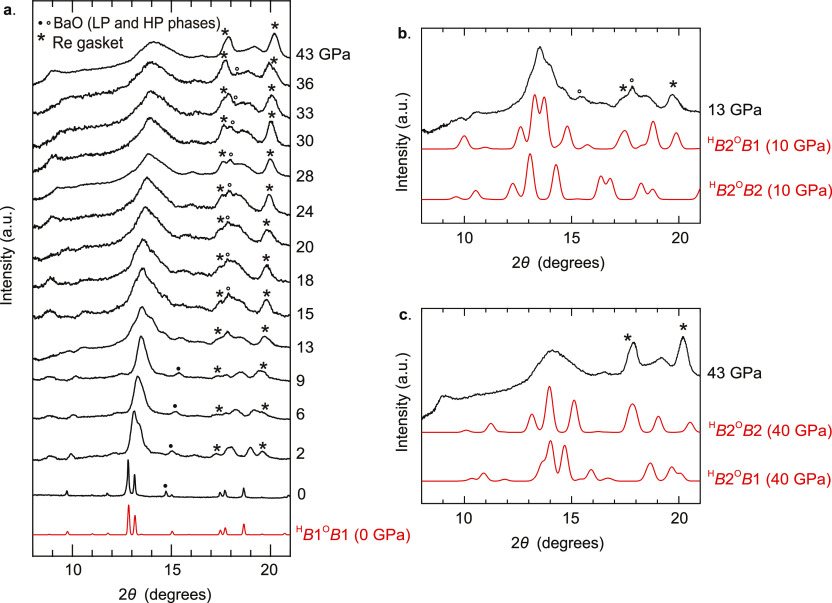
(a) Powder XRD patterns of Ba_2_YHO_3_ at room temperature up to 43 GPa. The 0 GPa pattern is reproduced from ref (18). Note that the original pattern was collected using Cu Kα radiation and has been converted here to a Mo Kα pattern. The simulated pattern of the ^H^*B*1^O^*B*1 structure at 0 GPa is shown for comparison. (b) Comparison of the experimental pattern at 13 GPa and calculated patterns (^H^*B*2^O^*B*1 and ^H^*B*2^O^*B*2) at 10 GPa. (c) Comparison of the experimental pattern at 43 GPa and calculated patterns (^H^*B*2^O^*B*2 and ^H^*B*2^O^*B*1) at 40 GPa. * represents peaks from the Re gasket, and open (closed) circles represent peaks from the high (low)-pressure phase of the BaO impurity.

With a quantitative prediction of the pressure-dependent behavior of Ba_2_YHO_3_ in hand obtained via DFT, we now report experimental data that offer clear evidence for the existence of the intermediate phase with mixed rock salt/CsCl ordering across a wide pressure range. The results of high-pressure powder XRD measurements for Ba_2_YHO_3_ in the range 2–43 GPa are summarized in Figure 3a. The ambient-pressure (0 GPa) powder pattern^18^ is also shown for comparison to the predicted pattern of the DFT-optimized ^H^*B*1^O^*B*1 structure, with very close agreement between the two. At 2 GPa, the diffraction patterns can be indexed to a tetragonal *P*-centered unit cell (S.G.: *P*4/*nmm*), consistent with the previous report at ambient pressure.^18^ The lattice parameters at 2 GPa are slightly compressed relative to the ambient-pressure values [*a* = 4.3062(18) Å and *c* = 13.5797(69) Å vs 4.38035(3) and 13.82338(10) Å, respectively]. There are small amounts of BaO impurity that may be derived from the decomposition of Ba_2_YHO_3_ during the preparation for the high-pressure experiments. We note that BaO exhibits a structural transition at 11–18 GPa.^27^ As the pressure is increased to 9 GPa, the 2θ reflection peaks broaden and shift to higher angles (Figure 3a), and the lattice parameters contract. Excellent agreement is found between the measured and calculated lattice parameters in this pressure range, as shown in Figure S1 in the Supporting Information.

Just above 9 GPa, a distinct change in the diffraction pattern occurs, suggesting the occurrence of a structural transition. The main peaks around 2θ ∼ 13° appear to split, and the high-angle intensities increase. These features indicate symmetry reduction, consistent with the transition from the tetragonal ^H^*B*1^O^*B*1 cell to the orthorhombic cell ^H^*B*2^O^*B*1, which our DFT calculations predicted at around 15 GPa. Comparison between the experimental pattern and the simulated pattern for the ^H^*B*2^O^*B*1 structure (Figure 3b) confirms that this assignment is consistent with the data, though the peak broadening precludes direct identification of the lattice system. Furthermore, the calculated diffraction pattern of the ^H^*B*2^O^*B*2 structure matches the experimental pattern much less closely than that of the ^H^*B*2^O^*B*1 structure, confirming that the low-pressure *B*1–*B*2 transition occurs in the H–Ba rock salt layer only (Figure 3b). The measured transition pressure of 9–13 GPa is marginally lower than the computed value, consistent with studies on related systems.^11^ To analyze further details of the transition, we carried out high-resolution synchrotron XRD measurements up to 23 GPa (Figure S2 in the Supporting Information), which reproduced the transition around 13 GPa. However, peak broadening was again observed above 5 GPa, which obscures the details of the structure under high pressure. This peak broadening may be derived from discrepancies between the compressibilities of the hydride and oxide layers,^11,14^ which enhances the strain in the structure as the pressure increases.

Our calculations led us to expect another transition in the O–Ba layer above 30 GPa, leading to the ^H^*B*2^O^*B*2 cell, but experimental evidence for this is less conclusive. Increasing the pressure above 13 GPa results in further broadening of the peak pattern (Figure 3a) and the intensity of peaks in the high 2θ region increase, notably at 18 and 20°. The simulated pattern of the ^H^*B*2^O^*B*2 phase matches the shape of the experimental pattern at 43 GPa (Figure 3c), but unfortunately, the 18–20° window of 2θ where the patterns of the ^H^*B*2^O^*B*1 and ^H^*B*2^O^*B*2 phases differ most obviously is obscured by peaks arising from the Re gasket. This, along with the generally low resolution that is typical of such high pressure, makes it difficult to draw any firm conclusions about the existence of the second transition in the measured pressure range.

So far we have considered transitions between crystalline phases, comparable to the behavior of other Ruddlesden−Popper phases. However, the substantial peak broadening observed at higher pressures, in both lab-based and synchrotron XRD, could also indicate the presence of an amorphous phase containing a mixture of rock salt and CsCl layers. This may equally be described as disorder in the form of stacking faults in the ideal crystalline phases. Amorphous phases may arise in Ba_2_YHO_3_, but not other RP phases, because of the lower symmetry, or the low electrostatic energy of the H-Ba layer which leads to the low critical pressure of the first transition. Nevertheless, we believe that the four crystalline phases described in Figure 2 are the limiting possibilities from which any amorphous phases will likely be composed, so we will explore these structures in detail in our computational investigation.

### Analysis of Structural Changes

**Figure 4:**
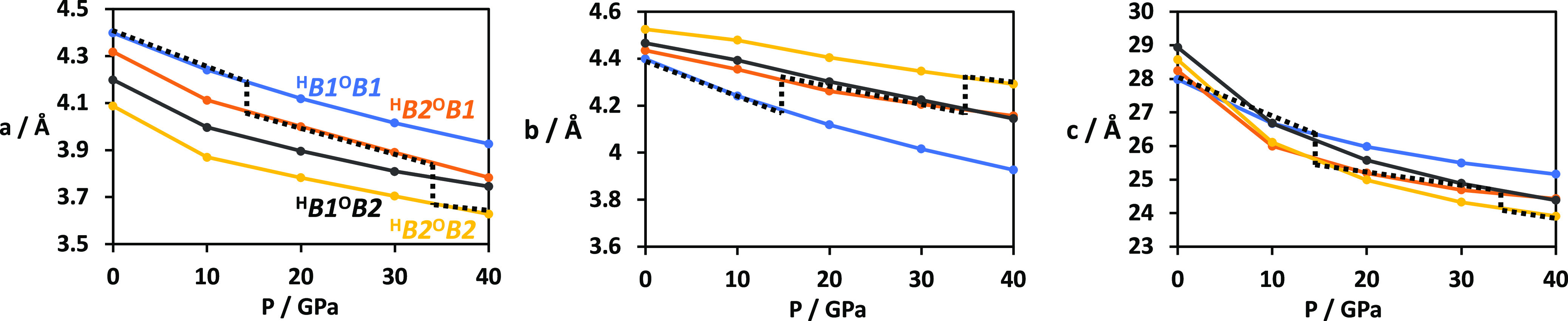
Lattice parameters of cells of Ba_2_YHO_3_ as a function of pressure. Blue lines refer to the ^H^*B*1^O^*B*1 cell, orange to ^H^*B*2^O^*B*1, black to ^H^*B*1^O^*B*2, and yellow to ^H^*B*2^O^*B*2. Dashed lines track the lowest enthalpy structure as the pressure changes, marking the predicted value of each lattice parameter of an experimental sample at any given pressure.

Due to the intrinsic limitations of high-pressure XRD, we have not been able to extract structural parameters of Ba_2_YHO_3_, such as lattice parameters or atomic positions, from our measurements. However, the close match between the simulated spectrum of the ^H^*B*2^O^*B*1 phase and the experimental data around 13 GPa indicates that this is indeed the phase present, and we now proceed to analyze the computed structures at the atomic level to understand in more detail the origins of the pressure-dependent behavior. We have plotted the lattice parameters for all four structures as a function of pressure in Figure 4. The dashed lines show the predicted lattice parameters at any given pressure by following the minimum-enthalpy structure at that pressure, with jumps from ^H^*B*1^O^*B*1 to ^H^*B*2^O^*B*1 at 15 GPa and from ^H^*B*2^O^*B*1 to ^H^*B*2^O^*B*2 at 35 GPa. This computational experiment allows us to explore the behavior of all possible cells at all pressures, providing insights unavailable from experimental results alone as any real experiment can probe only the changes along the dashed line.

The most dramatic pressure dependence is found along the *c* direction, where, at 0 GPa, the all-rock salt (^H^*B*1^O^*B*1) cell has the smallest lattice parameter, some 0.3 Å below ^H^*B*2^O^*B*1 and 0.6 Å below that for the all-CsCl alternative (^H^*B*2^O^*B*2). However, only a very slight increase in pressure is required to reverse this order, and by 10 GPa, the ^H^*B*2^O^*B*2 cell is the most compressed. The trends in *a* and *b* should be examined together as these are identical in the ^H^*B*1^O^*B*1 cell but are rendered inequivalent by any *B*1–*B*2 transition. The transition shifts the perovskite blocks by *a*/2, changing the ordering in the H–Ba or O–Ba layers such that like ions in these regions now have equal *a* coordinates, repelling each other more strongly along *b*. This is why the compressibility of *b* decreases with successive *B*1–*B*2 transitions while that of *a* increases.

**Figure 5:**
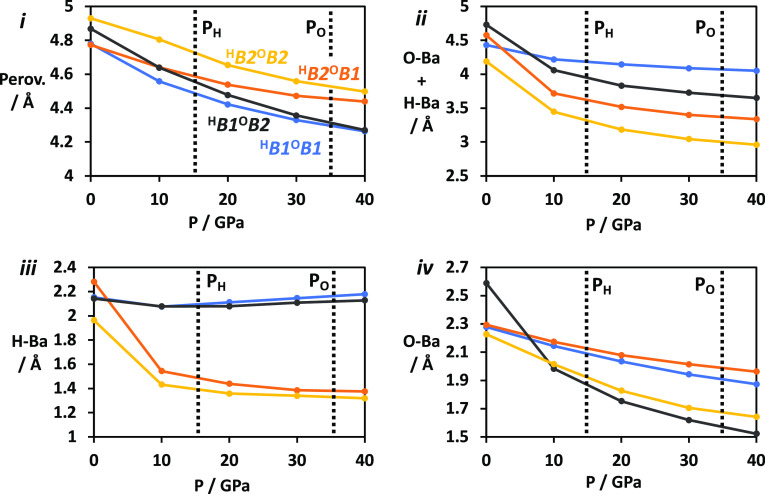
Compressibility of Ba_2_YHO_3_ along *c* in different regions of the unit cell. (i) Perovskite layer. (ii) Sum of O–Ba and H–Ba layers. (iii) H–Ba layer. (iv) O–Ba layer. Vertical dashed lines mark calculated *B*1–*B*2 transition pressures, with *P*_H_ and *P*_O_ denoting transitions in the H–Ba and O–Ba layers, respectively.

**Figure 6:**
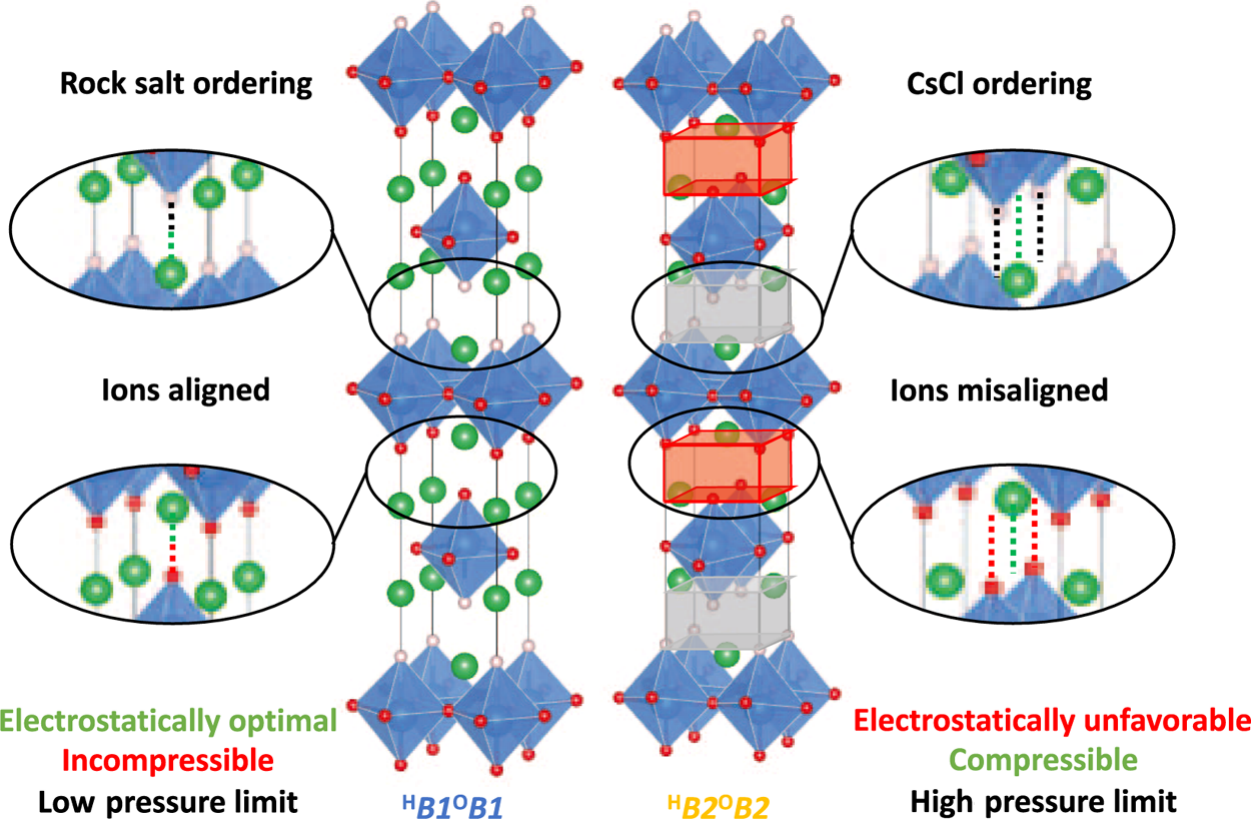
Unit cells of Ba_2_YHO_3_ detailing rock salt and CsCl orderings of H–Ba and O–Ba regions.

The trends in *c* indicate that the CsCl blocks provide the greatest compressibility, so to explore this further, we have plotted the widths of the structural blocks (shown in Figure 2) against pressure in Figure 5. Figure 5i shows the width of the perovskite block (“Perov.”), while Figure 5(ii) shows the sum of the O–Ba and H–Ba regions. The *c* lattice parameter is twice the sum of these components, so all factors affecting the compressibility of *c* are captured here. We can now see the far higher compressibility of the O–Ba and H–Ba blocks over the perovskite blocks—note the different length scales—for all cells possessing at least one *B*2-ordered layer. The H–Ba block is highly compressible for the ^H^*B*2^O^*B*1 and ^H^*B*2^O^*B*2 phases, while the O–Ba block is compressible for the ^H^*B*1^O^*B*2 and ^H^*B*2^O^*B*2 phases. The microscopic origins of the compressibility of the *B*2 layers are depicted in Figure 6 and are discussed above. The O–Ba and H–Ba blocks also give rise to the greatest difference between the cells, while there is little absolute variation in the widths of the perovskite blocks at any pressure (Figure 5i), which is unsurprising since the cells only differ in the ion ordering in the O–Ba and H–Ba regions. Above 10 GPa, the ordering of perovskite block widths is the reverse of the O/H–Ba widths. The perovskite blocks are all structurally identical, so this trend most likely appears because the cells preferentially compress the O/H–Ba regions, with changes to the perovskite blocks occurring only when the O/H–Ba regions incur more severe repulsive penalties. The ^H^*B*2^O^*B*2 cell, the most stable in the high-pressure limit, in fact has the largest perovskite block, indicating that the *B*1–*B*2 transitions in the H/O–Ba regions allow for a release of strain in compressed H/O–Y bonds. The width of the perovskite layer therefore changes by less than 0.2 Å between 0 GPa (where it is in the ^H^*B*1^O^*B*1 phase) and 40 GPa (in the ^H^*B*2^O^*B*2 phase).

We now turn to Figure 5c,d, showing the compressibilites of the H–Ba and O–Ba regions of each cell separately, to explain the remaining differences between the cells. Figure 6 shows these regions in more detail in both ordering patterns. Both graphs show two pairs of curves according to whether the relevant region of the cell is in rock salt or CsCl ordering. It is now unambiguous that CsCl ordering affords greater compressibility than rock salt ordering, confirming our interpretation of the trend in *c* compressibilities shown in Figure 4c and agreeing with previous results for Sr_2_VO_3_H. Figure 6 explains this geometrically, showing that rock salt ordering maximizes electrostatic attractions by bringing opposite ions into contact with one another by aligning them along *c*, so compression along *c* is resisted by repulsions between electronic cores. CsCl ordering disrupts this alignment, allowing unopposed *c* compression at the expense of electrostatic energy. Comparison of Figure 5c,d also shows that the H–Ba layer is made far more compressible by the *B*1–*B*2 transition than the O–Ba layer. This is because CsCl ordering is electrostatically unfavorable, as indicated in Figure 6, but this penalty is less severe in the H–Ba layer because the monovalent H^–^ generates smaller electrostatic energy contributions than the divalent O^2–^. The dramatic compression of the CsCl-ordered H–Ba blocks relative to their rock salt counterparts from 0 to 10 GPa ultimately leads to the first phase transition at 15 GPa, while the greater similarity in compressibilities between ordering patterns in the O–Ba layer means that this transition is enthalpically favored only above 35 GPa.

### Summary and Conclusions

In this paper, we have explored the behavior of Ba_2_YHO_3_, an anion-ordered oxyhydride, under high pressures. At ambient pressures, this adopts an RP structure with perovskite layers separated by Ba–O/H layers with rock salt ordering; unlike the closely related compound Ba_2_ScHO_3_, the hydride and oxide ions in the rock salt layer are ordered into separate regions of the unit cell. Initial studies using DFT identified the possible existence of an intermediate phase where the rock salt ordering switches to CsCl-type ordering (a *B*1–*B*2 transition) in the hydride-rich layer only, the transition occurring at a relatively accessible pressure of 15 GPa. Subsequent powder XRD measurements confirmed that this transition does indeed occur in the window between 9 and 13 GPa. The DFT calculations give access to the details of this transition at an atomic level, from which it becomes clear that the low critical pressure for the transition in the hydride-rich layer stems from the relatively weak H^–^–H^–^ repulsions, which allow for large compressibility in this region. The stronger O^2–^–O^2–^ repulsions in the oxide-rich layer, in contrast, resist compression and shift the critical pressure for the oxide layer above 30 GPa. The result is a wide window of stability for the new intermediate phase with mixed rock salt/CsCl ordering. The hydride-rich layer represents a “crumple zone” in the crystal, absorbing most of the impact of increasing pressure up to 30 GPa, allowing the perovskite layers to remain almost invariant to pressure across a remarkably wide pressure window. We hope that a deeper understanding of anion ordering and the influence of pressure will lead, ultimately, to the design of novel hydride ion conductors.

## Supporting Information Available

Geometries of all optimized structures in the VASP POSCAR format (ZIP)
Comparison of the measured and calculated lattice parameters and powder XRD patterns of Ba_2_YHO_3_(PDF)
