# Supplementary material for: Sequential Pressure-Induced B1–B2 Transitions in the Anion-Ordered Oxyhydride Ba2YHO3
Source: Inorg Chem. 2022 Apr 22;61(18):7043–50. doi: 10.1021/acs.inorgchem.2c00465 (PMC9092455; doi:10.1021/acs.inorgchem.2c00465)
Supplement: Supplementary file 2 — ic2c00465_si_002.pdf [file ic2c00465_si_002.pdf]

Supporting Information

Sequential pressure-induced $B1-B2$ transitions in the anion-ordered oxyhydride Ba_2YHO_3

Harry W.T. Morgan[1,2], Takafumi Yamamoto[3], Takumi Nishikubo[3,4], Takuya Ohmi[3], Takehiro Koike[3], Yuri Sakai[3,4], Masakai Azuma[3,4], Hirofumi Ishii[5], Genki Kobayashi[6,7], John E. McGrady[2]

[1]Department of Chemistry and Biochemistry, University of California, Los Angeles, Los Angeles, California 90095-1569, United States
[2]Department of Chemistry, University of Oxford, South Parks Road, Oxford OX1 3QR, United Kingdom
[3]Laboratory for Materials and Structures, Tokyo Institute of Technology, Yokohama, Kanagawa, 226-8503, Japan
[4]Kanagawa Institute of Industrial Science and Technology, Ebina 243-0435, Japan
[5]National Synchrotron Radiation Research Center, Hsinchu 30076, Taiwan [6]Department of Materials Molecular Science, Institute for Molecular Science, 38 Nishigonaka, Myodaiji, Okazaki, Aichi 444-8585, Japan
[7]SOKENDAI (The Graduate University for Advanced Studies), 38 Nishigonaka, Myodaiji, Okazaki, Aichi 444-8585, Japan

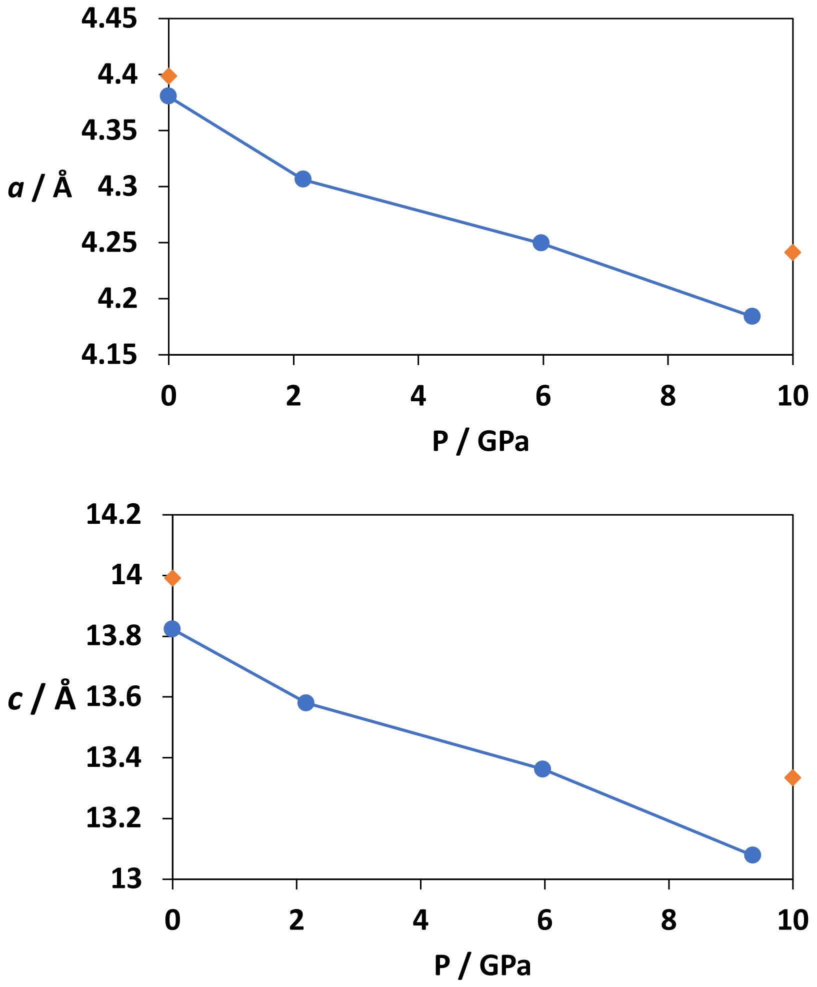

Figure S1: Comparison between lattice parameters measured by X-ray diffraction (blue line) and computed (orange points) of the ambient-pressure ${}^{\mathrm{H}}B1{}^{\mathrm{O}}B1$ structure of Ba_2YHO_3 between 0 and 10 GPa.

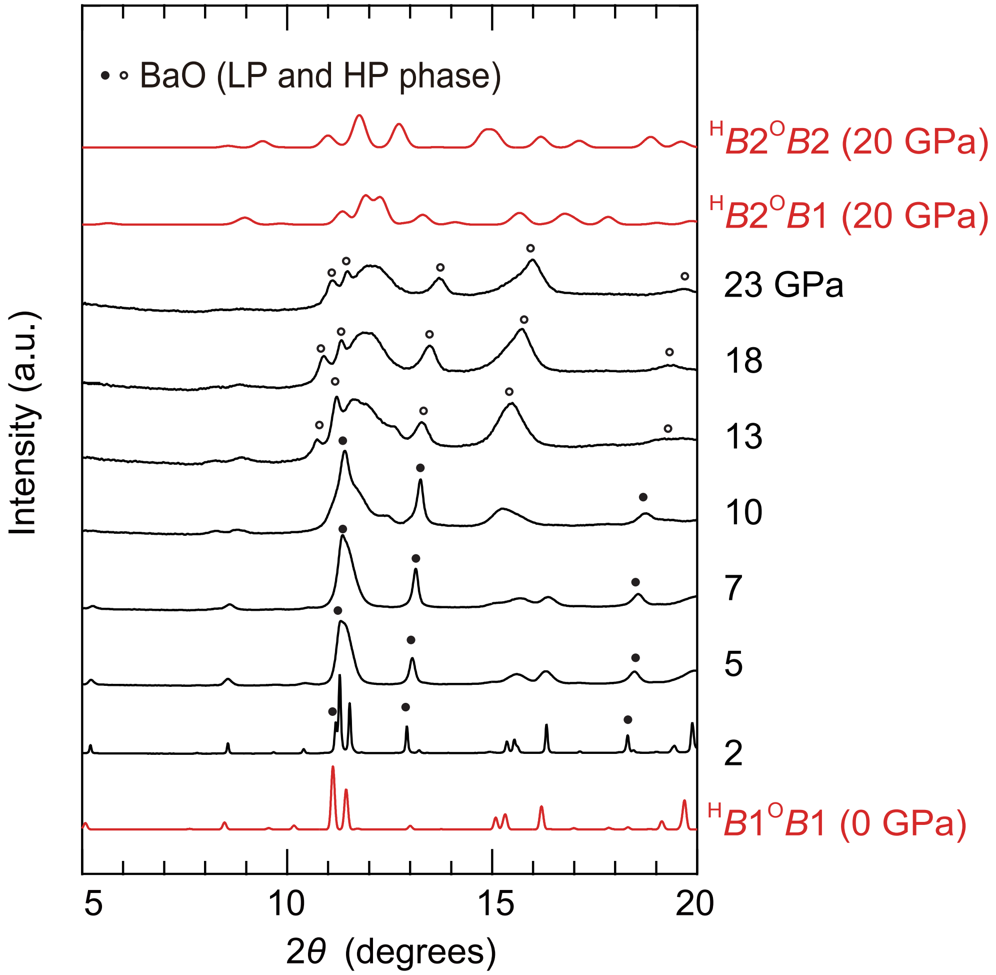

Figure S2: Synchrotron powder X-ray diffraction patterns for Ba_2YHO_3. Simulated patterns of the ${}^{\mathrm{H}}B1{}^{\mathrm{O}}B1$ phase at 0 GPa and the ${}^{\mathrm{H}}B2{}^{\mathrm{O}}B1$ and ${}^{\mathrm{H}}B2{}^{\mathrm{O}}B2$ phases at 20 GPa are shown for comparison.
